# Remodeling of Leaf Cellular Glycerolipid Composition under Drought and Re-hydration Conditions in Grasses from the *Lolium-Festuca* Complex

**DOI:** 10.3389/fpls.2016.01027

**Published:** 2016-07-19

**Authors:** Dawid Perlikowski, Sylwia Kierszniowska, Aneta Sawikowska, Paweł Krajewski, Marcin Rapacz, Änne Eckhardt, Arkadiusz Kosmala

**Affiliations:** ^1^Department of Environmental Stress Biology, Institute of Plant Genetics, Polish Academy of SciencesPoznan, Poland; ^2^Max Planck Institute of Molecular Plant PhysiologyPotsdam, Germany; ^3^Department of Biometry and Bioinformatics, Institute of Plant Genetics, Polish Academy of SciencesPoznan, Poland; ^4^Department of Plant Physiology, University of Agriculture in KrakowKrakow, Poland

**Keywords:** cell membranes, drought tolerance, *Festuca arundinacea*, grasses, lipidome profiling, *Lolium multiflorum*

## Abstract

Drought tolerant plant genotypes are able to maintain stability and integrity of cellular membranes in unfavorable conditions, and to regenerate damaged membranes after stress cessation. The profiling of cellular glycerolipids during drought stress performed on model species such as *Arabidopsis thaliana* does not fully cover the picture of lipidome in monocots, including grasses. Herein, two closely related introgression genotypes of *Lolium multiflorum* (Italian ryegrass) × *Festuca arundinacea* (tall fescue) were used as a model for other grass species to describe lipid rearrangements during drought and re-hydration. The genotypes differed in their level of photosynthetic capacity during drought, and in their capacity for membrane regeneration after stress cessation. A total of 120 lipids, comprising the classes of monogalactosyldiacyloglycerol, digalactosyldiacyloglycerol, sulfoquinovosyldiacylglycerol, phosphatidylglycerol, phosphatidylcholine, phosphatidylethanolamine, phosphatidylserine, phosphatidylinositol, diacylglicerol, and triacylglicerol, were analyzed. The results clearly showed that water deficit had a significant impact on lipid metabolism in studied forage grasses. It was revealed that structural and metabolic lipid species changed their abundance during drought and re-watering periods and some crucial genotype-dependent differences were also observed. The introgression genotype characterized by an ability to regenerate membranes after re-hydration demonstrated a higher accumulation level of most chloroplast and numerous extra-chloroplast membrane lipid species at the beginning of drought. Furthermore, this genotype also revealed a significant reduction in the accumulation of most chloroplast lipids after re-hydration, compared with the other introgression genotype without the capacity for membrane regeneration. The potential influence of observed lipidomic alterations on a cellular membrane stability and photosynthetic capacity, are discussed.

**HIGHLIGHTS**

- A higher drought tolerance of grasses could be associated with an earlier lipidome response to a stress signal and with a membrane regeneration after stress cessation accompanied by a turnover of chloroplast lipids

## Introduction

Water deficit, frequently occurring in a temperate climate, is one of the main abiotic factors that have a negative impact on plant metabolism, resulting in a lower plant yield (Bray et al., 2000; Yu and Li, 2014). The species adapted to variable ambient conditions are able to create numerous morphological and metabolic modifications which help them to survive periods of drought and to restore a proper cell functioning following stress cessation (Torres-Franklin et al., 2007). It has been widely confirmed that drought tolerant species can maintain a stability and integrity of cellular biological membranes in harmful conditions, while drought susceptible species suffer severe and usually irreversible cell damage, mainly due to a degradation of cellular membranes (Yu and Li, 2014).

Biological membranes are fundamental to sustaining the biological processes of living organisms, including plants. They act as a selective barrier responsible for triggering signal transduction as the first response to stress conditions (Heidarvand and Maali-Amiri, 2010). Dehydration, as well as subsequent re-hydration, drastically affects plant cell turgor, causing mechanical damage to cellular membranes (Wolfe and Bryant, 1999). Furthermore, water deficit interrupts electron transport during the light photosynthetic phase, increasing the production of reactive oxygen species and the activation of hydrolytic enzymes, and thereby initiating damage of key biological molecules, especially proteins, nucleic acids, and membrane lipids (Mittler, 2002). To properly adapt to stressful conditions a series of events in plant cells is required and this also involves remodeling of membrane lipid and fatty acid (FA) composition (Heidarvand and Maali-Amiri, 2010). Glycerolipids are the predominant group of membrane lipids. Due to the nature of a hydrophilic head, they can be further divided into two major categories: (*i*) phosphoglycerolipids, which are the main components of extra-chloroplast membranes (Moore, 1982), and (*ii*) galactolipids, which are the main building blocks of plant chloroplasts (Hölzl and Dörmann, 2007). The balance between the bilayer forming galactolipids, such as the digalactosyldiacyloglycerol (DGDG), and non-bilayer forming monogalactosyldiacyloglycerol (MGDG), together with a sufficient content of acidic sulfoquinovosyldiacylglycerol (SQDG) and phosphatidylglycerol (PG) in the chloroplast membranes is required to maintain membrane stability and the activities of key proteins associated with the photosynthesis (Páli et al., 2003; Hölzl and Dörmann, 2007). Similarly, in extra-chloroplast membranes the proportions between two most abundant classes of phosphoglycerolipids, bilayer forming phosphatidylcholine (PC), and non-bilayer forming phosphatidylethanolamine (PE), are crucial for their stability (Norberg and Liljenberg, 1991; Larsson et al., 2006). Less abundant extra-chloroplast phospholipids are phosphatidylserine (PS) and phosphatidylinositol (PI) classes, playing a significant role in biosynthesis of other lipids and stress signaling (Larsson et al., 2006; Liu et al., 2013). Leaves also contain lipids that have other than just structural functions, such as diacylglycerol (DAG) or triacylglycerol (TAG) lipids (Lin and Oliver, 2008).

Modifications of cellular membranes lipid composition during water deficit, in order to maintain their integrity and fluidity, have already been observed in plants (Repellin et al., 1997; Gigon et al., 2004; Torres-Franklin et al., 2007). These studies showed that the process of membrane lipid composition remodeling comprises two phenomena. The first one involves the changes of polar lipid head group proportions (Yu and Li, 2014). As shown in the work on *Arabidopsis thaliana* (thale cress) the decrease in the MGDG:DGDG ratio in the plastid membranes led to an increase of plant tolerance to drought or low temperature stresses (Gigon et al., 2004; Degenkolbe et al., 2012). A similar dependence was also observed in the case of the plasma membrane, where the lipid bilayer integrity was affected by a change in the proportion of PC and PE species (Larsson et al., 2006). The second type of membrane composition remodeling includes changes in the saturation level and chain length of FA forming glycerolipid non-polar tails. Highly saturated FA chains reduce membrane fluidity, while those polyunsaturated increase (Gigon et al., 2004; Yu and Li, 2014). These relations have high impact on maintaining transmembrane protein functions and photosystem activities (Quinn and Williams, 1983; Quartacci et al., 2000). However, plant survival following drought depends not only on their ability to protect key cell components against harmful factors, but also on their ability to restore the functions of cell biological structures during plant recovery in non-stressed conditions (Torres-Franklin et al., 2007). Most of our knowledge on the profiling of crucial metabolites, including cellular membrane lipids during stress conditions such as drought (e.g., Gigon et al., 2004) and low temperature (e.g., Uemura et al., 1995) derives from research on model species, especially *A. thaliana*. However, this research does not fully cover the picture of lipidome alterations in monocots, including grasses (Foito et al., 2009).

*Festuca arundinacea* (tall fescue) expresses relatively high levels of tolerance to abiotic and biotic stress conditions, including water deficit (Kosmala et al., 2007, 2012), while *Lolium multiflorum* (Italian ryegrass) is characterized by a relatively high yielding capacity and digestibility but significantly low levels of stress tolerance (Humphreys and Thomas, 1993). *L. multiflorum* and *F. arundinacea* hybridization enables the assembly of complementary characters of both species within a single genotype (Humphreys and Thomas, 1993; Humphreys and Pašakinskienė, 1996; Kosmala et al., 2007). Herein, two closely related introgression forms of *L. multiflorum* × *F. arundinacea* were used as models for the other grass species to decipher in detail a remodeling process of cellular glycerolipd composition during water deficit conditions and further re-hydration. We hypothesize that lipid metabolism during drought and re-watering might have a great impact on the ability to regenerate cellular membranes after stress cessation, and on photosynthetic performance during drought. These two traits were differentially expressed between the studied here introgression forms. Thus, the main objectives of this study were: (*i*) identification of leaf metabolic and membrane lipids in two analyzed introgression forms; (*ii*) analysis of identified lipids composition with respect to FA chain lengths and unsaturation levels; and (*iii*) comparative analysis of identified lipid accumulation levels between the analyzed introgression forms. To the best of our knowledge this is the first paper demonstrating comprehensive research on cellular glycerolipids profiling during drought conditions in forage grasses.

## Materials and methods

### Plant materials

Plant materials applied in the profiling of cellular glycerolipids involved two *L. multiflorum/F. arundinacea* introgression forms (genotypes 4/10 and 7/6), developed and selected earlier as described by Perlikowski et al. (2014). These two forms were obtained after four rounds of backcrossing of *L. multiflorum* (4x) × *F. arundinacea* (6x) hybrid to *L. multiflorum* (4x), and were selected from a larger population as the genotypes differing in the level of drought tolerance in the simulated drought conditions in the field. The genotype 4/10, with a higher level of drought tolerance, possessed a higher yielding potential (with respect to dry and fresh biomass) during 14 weeks of water deficit in the field, compared to the genotype 7/6, characterized by a lower level of tolerance. Further physiological observations during 11 days of simulated water deficit conditions in pots indicated additional differences existing between the analyzed genotypes. The electrolyte leakage (EL) parameter, describing the level of membrane stability, increased significantly on the last day (the 11th day) of drought application in the two genotypes, although after re-watering it returned to the values calculated for the conditions before drought initiation only in the genotype 4/10. Thus, significant differences between the analyzed forms, with reference to their capacity for membrane regeneration, were revealed 10 days after re-watering. Furthermore, CO_2_ assimilation [μmol(CO_2_) m^−1^s^−1^] was at the same level (values marked below with the same letter did not differ statistically at *P* = 0.05, according to Tukey HSD test) in both genotypes under the control conditions (11.48 ± 0.36*a* in the 4/10 and 11.01 ± 0.47*a* in the 7/6) but was significantly higher in the genotype 7/6 on the 11th day of drought treatment (7.42 ± 0.16*b*), compared to the genotype 4/10 (6.58 ± 0.17*c*). Under control conditions the CO_2_ assimilation rate was strongly dependent on the stomatal conductance, which was not observed in drought (Perlikowski et al., 2014). Also, no differences between the analyzed introgression forms were revealed with respect to chlorophyll fluorescence parameters, indicating the same photochemical performance of both genotypes in drought. Taken together, the differences in CO_2_ assimilation rate observed in the two genotypes were hypothesized to be associated with the Calvin cycle (Perlikowski et al., 2014).

The leaf tissue for the lipidomic work was collected from four biological replicates (clonal replicates) of each analyzed genotype during physiological analysis: before drought treatment (control), at three different time-points during drought treatment (after 3, 6, and 11 days of drought), and after 10 days of subsequent re-watering, and the tissue was then frozen in liquid nitrogen.

The experimental conditions during physiological analysis and plant tissue sampling for lipidome profiling were as described in details by Perlikowski et al. (2014) and Kosmala et al. (2012). Herein, a general design of the performed experiments is demonstrated. For the application of stress conditions the introgression forms were cloned in four pots (1.75 dm^3^) containing a sand:peat (1:3) mixture and placed in a growth chamber at a temperature of 22/17°C (16 h day/8 h night, light of 400 μmol(quanta) m^−2^ s^−1^, HPS “Agro” lamps, Philips, Brussels, Belgium), 30% relative air humidity and watering completed. During the whole experiment the water content in individual pots was controlled by daily weighing, and the content was equalized to the level observed in the clone that lost least water. The weight decrease in individual pots, and the mass of water added and corrected for the mass of the samples collected, were used for the calculation of both water uptake by individual plants (clones) as well as field water capacity changes during the whole experiment. The level of soil water content decreased from 63% of field water capacity observed in control conditions down to approximately 3% on the 11th day of stress duration. After 11 days of treatment plants were re-watered to the field water capacity, measured before the drought, and this level of water content in the soil was maintained by daily weighing and watering until the end of the experiment (until 10 days of re-watering).

### Lipid extraction and ultra performance liquid chromatography (UPLC)–mass spectrometry (MS) polar lipid measurement

Lipids were extracted using the protocol described by Hummel et al. (2011). The amount of 70 mg of frozen leaf tissue was placed in 2 ml Eppendorf tubes, frozen in liquid nitrogen and homogenized. Lipids were extracted from tissue with a precooled (−20°C) extraction mixture of methanol/methyl-tert-butyl-ether (1:3) with 1 μg ml^−1^ of Corticosterone (BioSolve UPLC grade), 0.5 μg ml^−1^ of 1,2-diheptadecanoyl-sn-glycero-3-phosphocholine (PC 34:0) (Avanti Polar Lipids, 850360P), 0.25 μg ml^−1^ of Ampicillin, 0.5 μg ml^−1^ of ^13^C Sorbitol. Samples were incubated for 30 min at 4°C on an orbital shaker, before incubating them for another 10 min in an ultrasonication bath at room temperature. The volume of 500 μl of UPLC grade water/methanol (3:1) mixture was added, mixed and centrifuged for 3 min at full speed at 4°C. After the phase separation, the upper organic phase was collected to a fresh 1.5 ml Eppendorf tube, dried under vacuum and re-suspend in 200 μl of isopropanol/water (7:3). The volume of 170 μl of mixture was transferred to a glass vial. Samples were measured using ultra high performance liquid chromatography coupled to an Exactive mass spectrometer (Thermo-Fisher). Lipids were separated on C8 reversed phase column (100 mm • 2.1 mm • 1.7 μm particles, Waters) at 40°C (Waters Acquity UPLC system, http://www.waters.com). The mobile phases included water with 1% 1 M NH4Ac, 0.1% acetic acid (UPLC grade; BioSolve) and acetonitrile/isopropanol (7:3) with 1% 1 M NH4Ac and 0.1% acetic acid (UPLC grade; BioSolve). The separation was run in linear 17 min gradient. The mass spectra were acquired using an Exactive mass spectrometer. Mass spectrometer was operating in full scan mode, covering a mass range from 100 to 1500 m/z with the resolution set to 10,000. The spectra were recorded from 1 to 17 min of the UPLC gradients (Hummel et al., 2011).

### Data analysis

**Figure 1:**
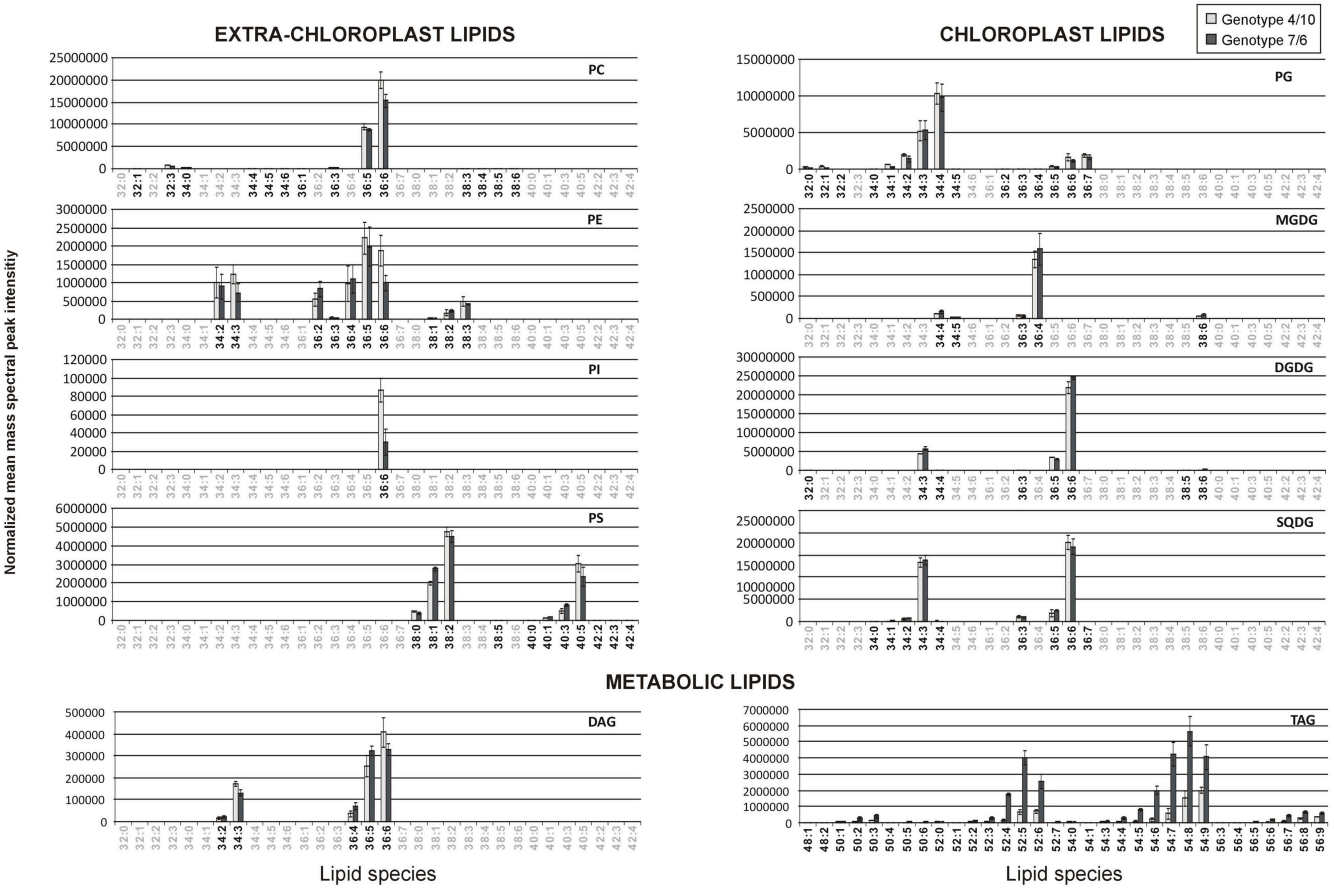
**The relative accumulation levels of analyzed lipid species within 10 main lipid classes**. The normalized mean mass spectral peak intensities with standard deviations are shown. Black numbers indicate the lipid species analyzed in the 4/10 and 7/6 genotypes, and gray numbers the species not analyzed. Lipid classes: DAG, diacylglycerol; DGDG, digalactosyldiacyloglycerol; MGDG, monogalactosyldiacyloglycerol; PC, phosphatidylcholine; PE, phosphatidylethanolamine; PG, phosphatidylglycerol; PI, phosphatidylinositol; PS, phosphatidylserine; SQDG, sulfoquinovosyldiacylglycerol; TAG, triacylglycerol.

The acquired chromatograms were processed by Refiner MS® (ver 7.5, GeneData http://www.genedata.com). The processing of MS data included peak detection and removal of fragmentation information, isotopic peaks, and chemical noise. The extracted peaks were annotated by querying against the in-house database that contains retention time and mass-to-charge ratio (m/z) information. The database contains lipids that were previously annotated based on their mass-to-charge ratio, supported by ^13^C isotope labeled *A. thaliana* (Giavalisco et al., 2011; Bromke et al., 2015) and a retention time shift of consecutive m/z values (Giavalisco et al., 2011; Degenkolbe et al., 2012; Bromke et al., 2015). Data on all the identified lipid analytes together with lipid database matchmaking were transferred to Excel for further filtering and normalization. The performed analysis allowed us to identify 165 lipid species in the analyzed introgression forms at five experimental time-points. Before further analysis, the lipids with a mean measured mass spectral peak intensity below 20,000, and present in less than 75% of all the analyzed samples (30 out of 40 samples), were rejected. Thus, for the statistical analysis a total of 120 lipid species were selected and subsequently divided into the classes with respect to the nature of their lipid head group (Figure 1). The detection method used allowed us to perform the comparative quantitative analysis only within the particular classes; such comparisons were not possible between the classes due to differences in lipid ionization levels, depending mainly on a type of lipid head group (Burgos et al., 2011). The measured mean mass spectral intensities and annotations for particular lipid species from four biological replicates are shown in the (Supplementary Table S1). The relative lipid intensities were normalized by dividing each lipid intensity in the sample by the median of lipid intensities in this sample. To retain peak intensity values for particular lipids, intensities in all the samples were multiplied by the median of intensities across all the samples before normalization. After the log_2_-transformation that made the data approximately normally distributed, the normalized intensities were used to evaluate the percent change of lipid species between the time-points. Principal component analysis (PCA) and biplots based on the correlation matrices were made using STATISTICA 10 software (StatSoft, Tulsa, OK). The PCA was performed for all the identified lipid classes, and for all the separate lipid species within a defined class. In these analyses an additional variable, EL, indicating a plasma membrane damage, was added. This physiological parameter had shown the clear differences between the analyzed genotypes during re-hydration conditions in our previous work (Perlikowski et al., 2014). Other statistical analyses, carried out using Genstat 17th Edition [VSN Int. Ltd., http://www.genstat.co.uk] and R 3.0.2. [http://www.r-project.org], included the two-way analysis of variance performed with genotype and time as experimental factors (time treated as repeated measurements). The significant effects of genotype, time, and genotype × time interaction were selected at *P* < 0.01. Differences between mean values at consecutive time points and the starting control values were computed, tested for significance using the Fisher's least significant difference test (at 5% level), and visualized using heatmaps. Means of log-transformed mass spectral peak intensities for lipid classes and lipid species with their standard errors, together with two-way ANOVA *P*-values, are presented in the (Supplementary Table S2). The Double Bond Index (DBI) was calculated from the log-transformed lipid intensities according to the formula (Orlova et al., 2003, modified):

(1) $$\text{DBI}=\frac{\begin{array}{l} (\sum_{i\in I_{1}}\text{i}+2\sum_{i\in I_{2}}\text{i}+3\sum_{i\in I_{3}}\text{i}+4\sum_{i\in I_{2}}\text{i}+5\sum_{i\in I_{5}}\text{i}\\ +\;6\sum_{i\in I_{6}}\text{i}+7\sum_{i\in I_{7}}\text{i}+8\sum_{i\in I_{8}}\text{i}+9\sum_{i\in I_{9}}\text{i}) \end{array}}{\left(100+\sum_{i\in I_{0}}\text{i}\right)}$$

where *Ij*–set of means of the log-transformed intensities of lipids with *j* = 0,1,…, 9 double bonds.

## Results

### A cellular glycerolipid composition with respect to chain length and unsaturation level

All the analyzed lipids identified in the two introgression forms could be assigned to three main groups depending on their sub-cellular location and physiological function (Figure 1). Chloroplast membrane lipids were represented by galactolipids, including the MDGD class (5 identified lipid species), one Lyso-MGDG lipid, the DGDG class (8 species) and one Lyso-DGDG lipid. The other chloroplast membrane lipids involved the SQDG class (8 species) and the PG class (19 species). The second group comprised extra-chloroplast membrane phospholipids represented by the PC class (15 lipid species), the PE class (10 species), the PS class (11 species) and the PI class (1 species). The third group involved metabolic lipids—the DAG class (5 species) and the TAG class (36 species). The identified lipid species within the particular classes showed a high degree of variation with respect to the length and unsaturation level of FA chains (Figure 1).

**Figure 2:**
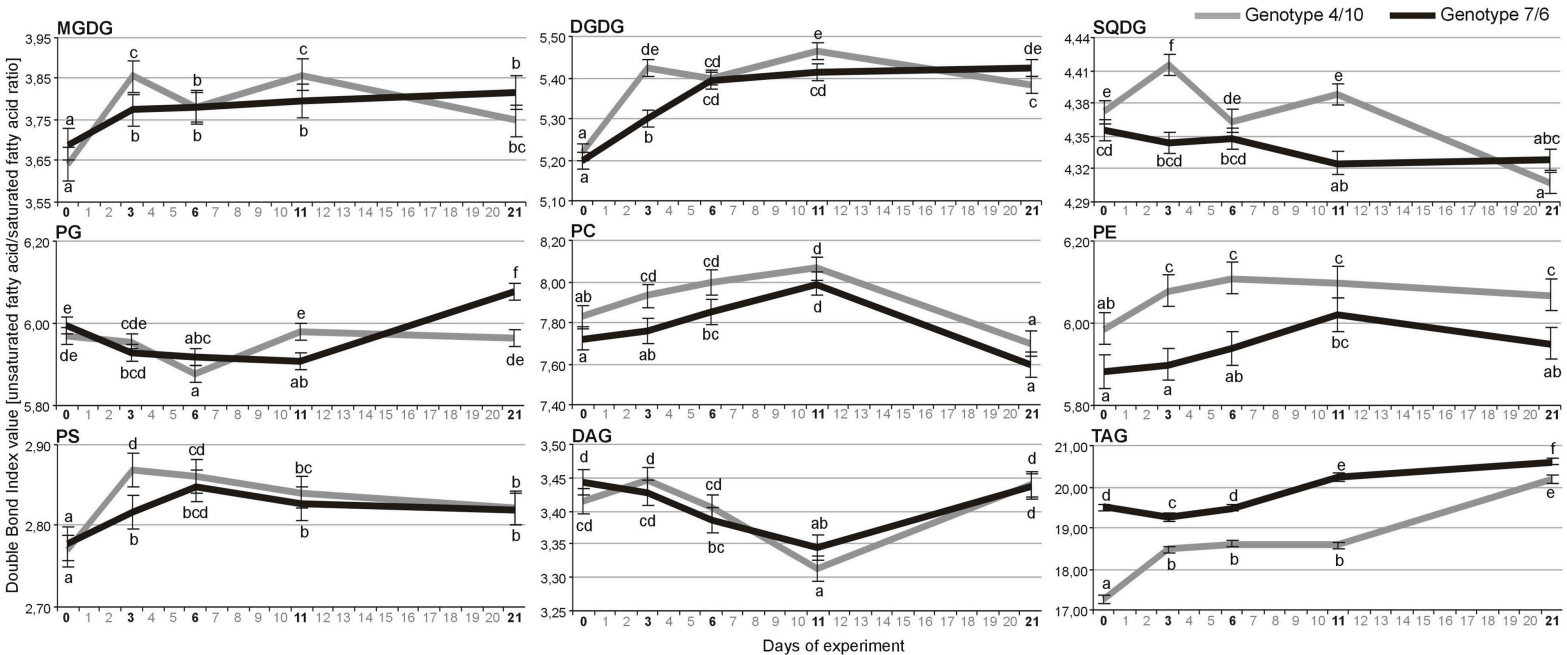
**The Double Bond Index (DBI) calculated for the pools of nine lipid classes with more than one identified individual lipid species**. Calculations were performed based on means of log-transformed mass spectra peak intensities measured for a particular lipid species within a lipid class at four time-points of experiment: after 3, 6, and 11 days of drought, and after 10 days of re-hydration (the 21st day of experiment) in the 4/10 and 7/6 genotypes. Error bars represent standard errors of the means. The letters indicate groups of means that do not differ significantly at a significance level of 0.05 (Fisher's LSD-test). Lipid classes denoted as in Figure 1.

Statistical analysis indicated that the extra-chloroplast lipid classes revealed only significant time-point differences in the DBI (Supplementary Table S2). The DBI level calculated for the main lipid classes (Figure 2) generally increased in both genotypes during drought and re-hydration for the main lipid types, including the MGDG, DGDG, PE, PS class, and storage TAG lipids (Figure 2). The significant differences in unsaturation level with respect to the interaction between the genotype and time-point were observed in the MGDG, DGDG, SQDG, PG, and TAG (Supplementary Table S2).

### The contribution of lipid classes to the functioning of analyzed genotypes during drought and re-hydration

**Figure 3:**
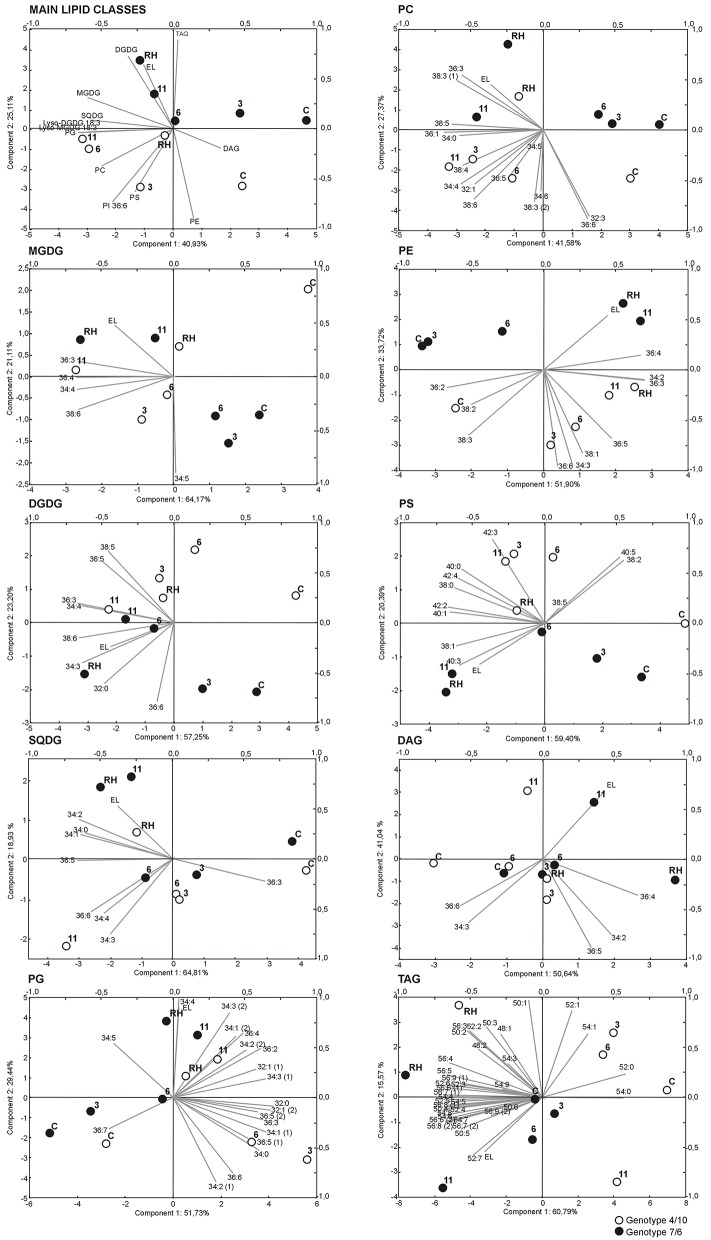
**Biplots of intensities of main lipid classes and particular analyzed lipid species, and electrolyte leakage values (EL), drawn using two principal components with the highest variance**. Black circles represent genotype 7/6, white circles - genotype 4/10. Time points are labeled by: **C**, control values; **3**, the third day of drought; **6**, the sixth day of drought; **11**, the 11th day of drought; **RH**, 10 days after re-hydration. Gray lines represent analyzed variables (lipid intensity, lipid class intensity, EL). Lipid classes denoted as in Figure 1.

The two-way analysis of variance for main lipid classes revealed four variables significantly discriminating the genotypes, six discriminating time points, and three expressing genotype x time point interaction (Supplementary Table S2). The biplot visualization (Figure 3) demonstrated that the time-point was the main differentiating factor, with a variability related to the first principal component (PC1), and with the largest role of the PG, MGDG, SQDG pools, and Lyso-MGDG 18:3 (as inferred from ANOVA, Supplementary Table S2), and the PCA loadings (Supplementary Table S3). The differences between genotypes were related mainly to the TAG pool and PI 36:6 lipid and were visualized by the PC2 (Supplementary Tables S2, S3). The significant interaction between genotypes and time-points was evident for the TAG, PG, MGDG pools (Supplementary Table S2), and in the multivariate biplot representation it was seen in the re-hydration process, but only for the PG and MGDG pools, where a partial return of drought-induced levels to the ones observed before drought treatment (“lipidome turnover”) was observed in the genotype 4/10 (Figure 3).

### A chloroplast membrane lipid composition

The detailed analysis prepared for individual chloroplast lipid species within classes MGDG, DGDG, SQDG, and PG revealed 14 variables significantly discriminating the genotypes, 34 discriminating time points, and 13 expressing genotype x time point interaction, which showed the largest dependence of the accumulation on time (Supplementary Table S2). This was also revealed in the biplot visualization with respect to the PC1. A significant return of lipid accumulation levels to the values in the control conditions was observed in the genotype 4/10 after re-hydration (Figure 3). This was shown especially for the MDGD and DGDG species with the most important contribution to this separation of MGDG 34:4, 36:4, 38:6 and DGDG 34:4, 36:5, 38:5, 38:6. The SQDG biplot presented a similar pattern in PC1 for the 36:5 and in PC2 for the 34:3, 34:4 and 36:6 SQDG species. According to the PG species accumulation level, the PC1 separated genotypes with respect to the third day of drought within a majority of PG lipid species, and indicated a partial return of their accumulation level in the genotype 4/10 after re-hydration to the values observed in the control conditions (Figure 3, Supplementary Table S3).

**Figure 4:**
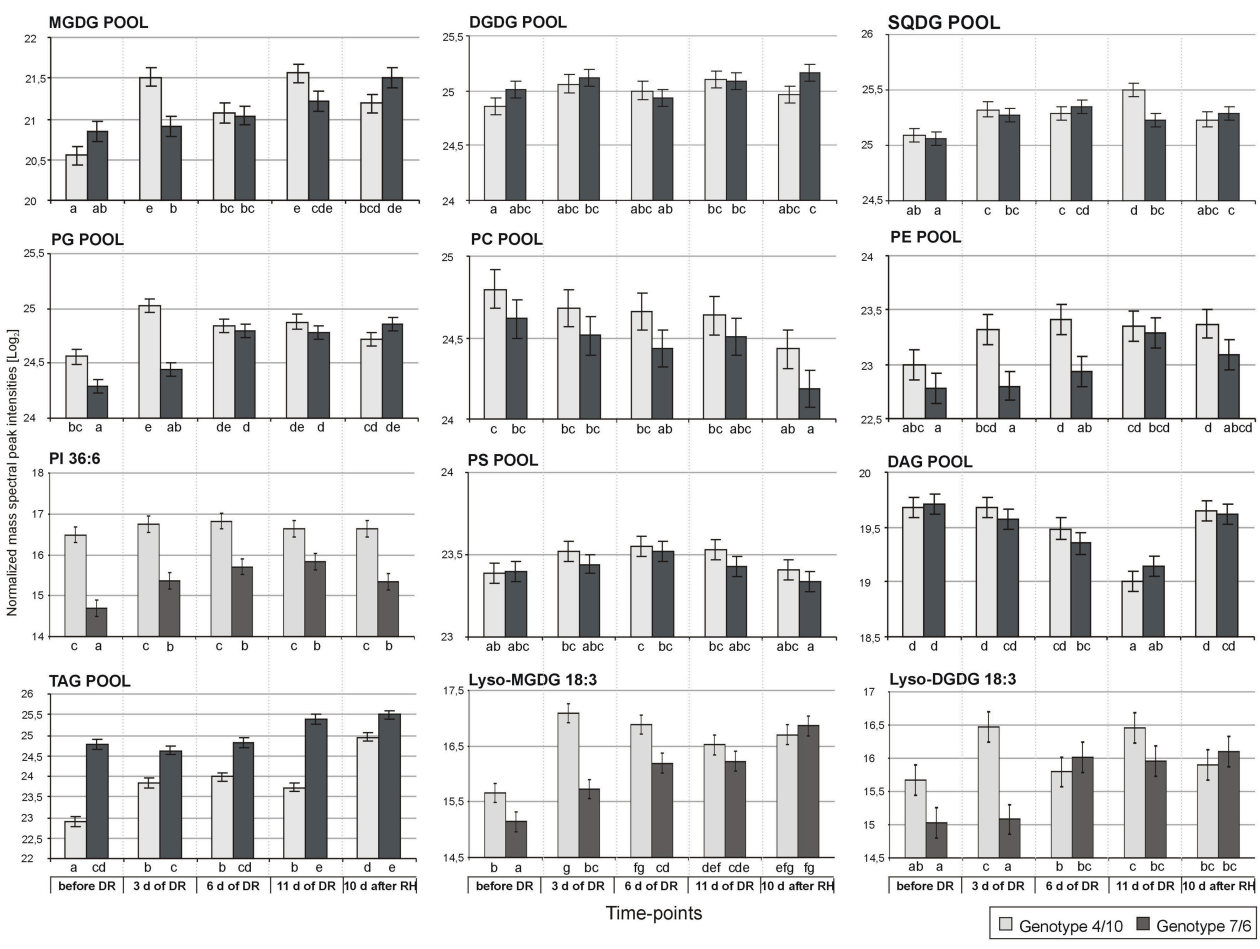
**The accumulation levels of analyzed lipid classes at five time-points of experiment: before drought, after 3, 6, and 11 days of drought (DR), and 10 days of re-hydration (RH) in the 4/10 and 7/6 genotypes**. The bars represent a mean value (over replications) for Log_2_ transformed sum of all the lipid species mass spectra peak intensities within a particular lipid class. Error bars represent standard errors of the means. The letters indicate groups of means that do not differ significantly at a significance level of 0.05 (Fisher's LSD-test). Lipid classes denoted as in Figure 1.

**Figure 5:**
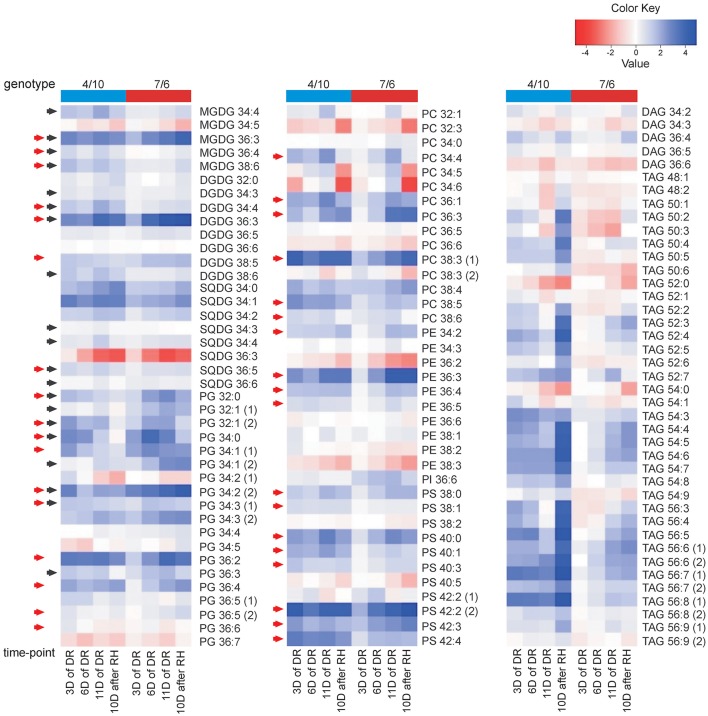
**The accumulation of lipid species at four time-points of experiment: after 3, 6, and 11 days (D) of drought (DR), and after 10 days of re-hydration (RH) in the 4/10 and 7/6 introgression forms**. The values shown are differences between mean Log_2_ transformed mass spectral peak intensities of particular lipid species at four time-points and control values; the values lower than the control are shown in shades of red and the values higher than the control are shown in shades of blue. The black arrows indicate chloroplast lipids exhibiting a return of accumulation between 11th day of drought and re-hydration to the levels observed before drought application in the 4/10 genotype, red arrows - lipids with a significantly higher accumulation level on the third day of drought in the genotype 4/10. Lipid classes denoted as in Figure 1.

An accumulation level of the MGDG pool was significantly higher, compared to the control conditions, during the whole drought and re-hydration period in the genotype 4/10, and on the 11th day of drought treatment and after re-watering in the genotype 7/6. The clear differences in the total amount of MGDG species between the analyzed genotypes were observed only on the third day of drought stress (Figure 4). However, the detailed accumulation profiles of particular MGDG species showed more differences between the experimental time-points and the analyzed genotypes (Supplementary Figure S1). A significant increase of accumulation levels at the beginning of drought period (the third day of drought stress), in comparison to the control, was observed in both genotypes for the MGDG 34:4, 38:6 and 36:3, but only in the genotype 4/10 for the MGDG 36:4. However, the genotype 4/10 accumulated a two fold higher amount of those lipid species, compared to the genotype 7/6 (Supplementary Table S4). After re-hydration, the genotype 4/10 also showed the reduced abundance of MGDG 34:4, 36:3, 36:4, and 38:6, compared to the 11th day of drought stress (Figure 5, Supplementary Figure S1). Furthermore, this tendency of abundance reduction after re-hydration in the genotype 4/10 was also observed for the total MGDG pool (Figure 4).

No significant differences with respect to a total DGDG pool were observed between the analyzed genotypes during the whole experiment (Figure 4). However, clear differences were observed with respect to the accumulation level of particular DGDG species. Five out of eight DGDG lipid species were accumulated to a higher degree in the 7/6 genotype under control conditions (Supplementary Figure S1). A significant increase of accumulation level for a majority of DGDG lipids under drought treatment, especially on the third day of drought was observed in the genotype 4/10. A similar tendency was also noticed in the genotype 7/6, but the increase was achieved more gradually. In the case of DGDG 34:3, 34:4, 36:3 and 38:6 a significant reduction of accumulation level in the genotype 4/10 during re-hydration, compared to the 11th day of drought was observed (Figure 5, Supplementary Figure S1).

The total pool of SQDG increased significantly during drought in both analyzed genotypes, compared to the control conditions, showing also a higher abundance on the 11th day of stress duration in the genotype 4/10. During re-hydration the SQDG pool decreased in the 4/10 to the value observed in the control and remained unchanged in the 7/6 genotype (Figure 4). In general, the increased abundance of the most SQDG lipid species during drought stress in both genotypes was observed. Significant differences between the analyzed genotypes in the level of accumulation of 34:3, 34:4, 36:3, 36:5, and 36:6 species were observed at some time-points during drought duration, but the lipid abundance was always higher in the genotype 4/10. A significant turnover of most lipid species was observed in the genotype 4/10 after re-hydration, compared to the 11th day of drought (Figure 5, Supplementary Figure S1).

The total pool of PG species showed a higher accumulation level during the whole drought period in the 4/10 genotype, and on the two last time-points of drought treatment in the 7/6 genotype (Figure 4). After re-hydration, the abundance of PG pool decreased to the control values in the genotype 4/10. Significant differences between the two genotypes existed before stress treatment, and after 3 days of drought, showing a higher accumulation level in 4/10 (Figure 4). Fifteen out of 19 PG species showed a significantly increased accumulation level during drought in both genotypes, but a different accumulation dynamic between the genotypes was noticed. The genotype 7/6 was in most cases characterized by a gradual increase of PG lipids accumulation during drought, while the genotype 4/10 showed very fast response to stress conditions, increasing levels of PG species accumulation already on the third day of water deficit. After re-hydration, a slight turnover of those lipids was observed, especially in the genotype 4/10 (Figure 5, Supplementary Figure S1).

### An extra-chloroplast membrane lipid composition

The analysis for the extra-chloroplast lipid species within classes revealed 15 variables significantly discriminating the genotypes, 28 discriminating time points, and none expressing genotype x time point interaction (Supplementary Table S2), which indicated that the time-point and genotype differences had the highest impact on the separation of PC, PE, and PS lipid species on the biplot visualization (Figure 3). The PC1 separated time-points of the experiment and showed almost no difference between the genotypes, except the third day of treatment. The individual lipid species with the highest loading values for the PC1 were, for the PC: 36:1, 38:5, for the PE: 34:2, 36:3, and 36:4 and for the PS: 38:0, 38:1, 40:1, 42:2 (2), and 42:4 species (Supplementary Table S3). Significant differences between the genotypes were observed in the accumulation of individual PE and PS species according to the PC2. Individual lipid species with the highest loading value for the PC2 were the 34:3, 36:6, 38:1 for the PE, the 40:0 and 40:5 for the PS, which were accumulated more highly in the genotype 4/10 than in the 7/6 (Supplementary Figure S1). A similar grouping was also observed in the case of PC 32:3, 36:6, 38:3 (2), and 38:6 species, with the exception of re-hydration time-point in 4/10 genotype (Figure 3).

No significant differences in the abundance of the PC pool between the genotypes and time points were observed, with the exception of its decrease after re-hydration (Figure 4). Most of the PC lipid species increased in accumulation during drought in both genotypes. However, for the PC 34:4, 36:1, 36:3, 38:3(1), 38:4, 38:5, and 38:6 species a higher accumulation level was observed in the genotype 4/10 at the beginning of drought, compared to the control conditions (Figure 5), and compared to the 7/6 genotype (Supplementary Figure S1). In the case of the 32:1, 32:3, 34:4, 34:5, 34:6, 36:6, and 38:6 species a significant reduction of their abundance after re-hydration was observed in both genotypes, compared to the 11th day of drought treatment (Supplementary Figure S1).

The PE pools showed an increased accumulation level on the sixth day of drought in the 4/10 genotype, compared to the control, and on the 11th day of drought in the 7/6 genotype. This accumulation level remained unchanged in both genotypes after re-hydration. During the two first time-points of drought stress duration a significantly higher abundance of PE pools in the 4/10 was noticed (Supplementary Figure S1). In the case of 34:3, 36:6, and 38:1 lipid species accumulation levels were rather constant throughout the whole experiment, but almost 2-fold higher in the genotype 4/10. The rest of PE lipid species, except 36:2, 38:2, and 38:3 lipids, showed more or less significant increase both in drought and after re-hydration. The 4/10 genotype was characterized by a significantly higher accumulation level of those lipid species on the third day of drought without further changes (Figure 5, Supplementary Figure S1).

Only one PI lipid species, 36:6 was identified here, and it revealed high differences in the accumulation profile between the analyzed genotypes. The genotype 4/10 was characterized by a significantly higher accumulation level of PI during the experiment, compared to the 7/6 genotype (Figure 4).

No significant change in the total pool of PS species was observed during the whole experiment, with no differences between the analyzed genotypes noticed (Figure 4). In a majority of identified PS species, including the 38:0, 38:1, 40:0, 40:1, 40:3, 42:2(2), 42:3, and 42:4 a similar pattern of accumulation level was observed. The genotype 7/6 revealed a gradual increase of lipid abundance during drought and the re-hydration period. On the other hand, the genotype 4/10 revealed a higher increase of accumulation levels of particular lipids at the beginning of the drought period, and their further constant levels until the end of the experiment (Figure 5, Supplementary Figure S1).

### A metabolic lipid composition

The total of the DAG species pool revealed a significant decrease during drought and a further increase after re-hydration in both analyzed genotypes (Figure 4). This tendency was also observed in most of particular DAG lipid species (Figure 5, Supplementary Figure S1). The statistical analysis performed for the TAG species revealed that 24 variables significantly discriminated the genotypes, 30 time-points, and 26 expressed genotype × time-point interaction (Supplementary Figure S1, Supplementary Table S2). The PCA revealed that the PC1 separated the genotypes in the control and on the third, sixth and 11th day of drought with respect to an accumulation of highly unsaturated lipid species (Figure 3, Supplementary Table S3). The accumulation of TAG revealed differences between the two genotypes, showing a higher abundance in the 7/6 genotype (Figure 4). The genotype 4/10 accumulated higher amounts of TAG during drought, and after re-watering these amounts were increased again. In the 7/6 genotype the TAG pool increased only after 11 days of drought (Figure 4, Supplementary Table S4). The highly saturated 52:0 and 54:0 species decreased during drought and recovery. The TAG species with a low level of unsaturation (48:1, 48:2, 50:1, 50:2, 50:3, 52:1, 52:2, and 54:1) showed a significant decrease after 11 days of dehydration and a further significant increase after re-watering in the 4/10 genotype. The genotype 7/6 revealed a slight but significant decrease of those lipids accumulation level in drought and a return to the control values after re-hydration. The TAG species with a medium unsaturation level (56:5, 52:3, 52:4, 52:5, 52:6, 56:3, and 56:4) in the genotype 4/10 showed a similar pattern of accumulation as in the previous group. On the other hand, in the genotype 7/6 their accumulation levels increased significantly after 11 days of drought and remained constant after re-watering. The highly unsaturated TAG species showed two fold higher increased accumulation level on the third day of drought in the genotype 4/10, compared to the control and after re-watering these amounts were doubled again. In the genotype 7/6, their accumulation level increased on the 11th day of drought (Figure 5, Supplementary Figure S1, Supplementary Table S4).

## Discussion

### A cellular glycerolipid composition with respect to unsaturation level

An increasing level of DBI in the PC, PE, PS, MGDG, DGDG, and TAG on the 11th day of drought was revealed in both *L. multiflorum/F. arundinacea* introgression forms (Figure 2). However, a significantly higher DBI of the majority lipid classes including the PC, PE, PS, MGDG, DGDG, and SQDG on the third day of drought and chloroplast lipids on the 11th day of drought was observed in the genotype 4/10, indicating a possible faster response of this genotype to changes in the environment. This phenomenon could be associated with the improved stress signaling in this introgression form. An increasing unsaturation level of polar lipids was reported previously during water deficit conditions with respect to plant drought tolerance level. Plants with a higher drought tolerance increased level of polyunsaturated FAs during drought, compared to the control (Monteiro de Paula et al., 1990; Repellin et al., 1997; Gigon et al., 2004; Torres-Franklin et al., 2007). It was also observed that polyunsaturated FAs play a significant role in maintaining the functionality of photosystems, and the activity of membrane-bound proteins (Quinn and Williams, 1983; Quartacci et al., 2000). An increased accumulation level of lipids rich in polyunsaturated FAs enhanced the tolerance of plants to desiccation (Li et al., 2014) or drought (Gigon et al., 2004) and improved a plant's capacity to recover after the re-hydration (Liu et al., 2013).

### A chloroplast membrane lipid composition

Galactolipids are synthesized in a chloroplast envelope and synthesis of their main members, the MGDG and DGDG, is strictly related because the MGDG lipids are precursors in DGDG synthesis (Awai et al., 2001; Kelly et al., 2003). In our investigations, the FA chain composition of DGDG class resembled those of MGDG class for only three lipid species (34:4, 36:3, and 38:6). However, the presence of another DGDG species with no corresponding MGDG species is striking. It is possible that the analyzed genotypes are characterized by a high activity of galactolipid:galactolipid galactostyltransferase (GGGT) (Kelly et al., 2003), while the synthesis of DGDG via DGD synthase plays a less significant role in these introgression forms (Hölzl and Dörmann, 2007).

Thylakoid membranes are highly conserved in relation to the proportions of particular chloroplastic lipid classes. The nature of membranes changes according to the ratio of non-bilayer to bilayer forming lipids (Hölzl and Dörmann, 2007). A composition of lipids in chloroplast membranes during water deficit conditions was previously reported to correspond with the level of plant tolerance to stress (Monteiro de Paula et al., 1990; Repellin et al., 1997; Yu and Li, 2014). Susceptible plants showed a degradation of galactolipids even in moderate drought conditions, while plants characterized by a higher level of tolerance showed constant or increased level of galactolipids even in very severe drought, especially within the DGDG class (Torres-Franklin et al., 2007), increasing the DGDG:MGDG ratio. In the present study, a majority of MGDG lipid species were characterized by a higher level of accumulation, especially on the 11th day of drought, compared to the control conditions in both genotypes. Interestingly, the pool of DGDG remained unchanged in both species. Earlier research performed on other plants including dicots (Monteiro de Paula et al., 1993; Repellin et al., 1997; Gigon et al., 2004; Yu and Li, 2014) and monocots such as *Hordeum vulgare* (barley) or *Triticum aestivum* (wheat) (Chetal et al., 1981) showed an increasing ratio of DGDG:MGDG during drought treatment, compared to the control, in more drought tolerant genotypes, which was not observed in this study.

The PG and SQDG anionic lipids located in thylakoids play an essential role in the stabilization of PSI and PSII activity in a wide range of environmental conditions (Sato et al., 2003, 2004; Kobayashi et al., 2015). The research conducted on *A. thaliana* mutants with a reduced level of these glycerolipids production revealed that the plants were characterized by a decrease in a chlorophyll content in leaves, reduced photosynthesis, changes in the chloroplast structure and impaired growth (Härtel et al., 1997; Jarvis et al., 2000). Previous studies describing plants response to drought conditions showed that tolerant genotypes of wheat and *Craterostigma plantagineum* accumulated the SQDG lipids during stress in contrast to susceptible genotypes (Quartacci et al., 1995; Gasulla et al., 2013). In the current work, many species of SQDG and PG lipids showed a higher accumulation level during drought in both genotypes, which could be associated with a stable chlorophyll fluorescence level observed in the two introgression forms under drought (Perlikowski et al., 2014). Furthermore, a low accumulation level of DGDG, with a simultaneous increased abundance of MGDG, did not seem to have impact on the photochemical performance in the two analyzed forms, as no significant reduction in chlorophyll fluorescence parameters was observed during the drought period (Perlikowski et al., 2014). However, it is worth mentioning that a higher accumulation level of many lipids from the SQDG and PG class were observed on the 11th day of drought in the 4/10 genotype with a lower photosynthetic capacity. Thus, these results confirm our earlier hypothesis that the higher efficiency of Calvin cycle under drought could be responsible for the better photosynthetic performance of genotype 7/6 on the 11th day of stress (Perlikowski et al., 2014). The other cellular factors, including those associated with a membrane stability and photochemical activity, seem to be less important for this phenomenon. However, this aspect of the research requires further study.

In the case of most MGDG and PG species, as well as particular lipids from the DGDG and SQDG classes, an earlier response to drought conditions was more visible in the genotype 4/10, compared to the genotype 7/6. These lipids started to increase their abundance on the third day of stress treatment in the 4/10 and this might be associated with a higher activity of galactolipid synthesis enzymes (Torres-Franklin et al., 2007) or more efficient stress signaling pathways (Liu et al., 2013; Okazaki and Saito, 2014) in that genotype. Such a strategy could be also crucial for a development of tolerance during prolonged stress treatment in the field conditions. In fact, as it had been demonstrated before, the genotype 4/10 revealed a higher yield potential during such a prolonged stress treatment, compared to the genotype 7/6 (Perlikowski et al., 2014). Here, after re-hydration in the genotype 4/10 a significant reduction in the accumulation levels of most MGDG, SQDG, and particular DGDG species was observed, compared to the 11th day of drought. This could be an effect of higher capacity of that genotype to regenerate after re-watering and to repair its damaged cellular membranes (Perlikowski et al., 2014).

### An extra-chloroplast membrane lipid composition

In extra-chloroplast membranes the main phospholipid compounds are the non-bilayer forming PE lipids and the highly abundant bilayer forming PC lipids. The balance between them is important for plants to preserve the membrane fixity (Larsson et al., 2006). Moreover, the PC lipids may serve as precursors for the synthesis of other glycerolipids (Ohlrogge and Browse, 1995). The PE species play a significant role in stress signaling pathways (Chapman, 2000). Earlier work conducted on *Avena sativa* (oat) showed that dehydration stress induced a decrease in the PC:PE ratio in the plasma membrane (Norberg and Liljenberg, 1991). It was suggested that changes in their ratio were related to *de novo* synthesis of PE which showed elevated abundance under drought conditions in *A. sativa*. Modifications of PC:PE ratio in drying plants might be associated with membrane flexibility, and the stability of membrane proteins. It was suggested that an increased level of PE in membranes of plants suffering from drought might help to reduce membrane damage during protoplast shrinkage, and to keep membrane enzymes active due to the lipid-protein interactions (Larsson et al., 2006).

The higher accumulation levels of selected PC and PE lipid species in the genotype 4/10 observed in our study at the beginning of drought may be associated with a postulated faster response of this genotype to changed environmental conditions. This strategy could be associated with a higher extra-chloroplast membrane stability of genotype 4/10. Moreover, a higher accumulation level of PE lipid pool and a decrease in an accumulation level of PC lipid pool in the genotype 4/10, most likely also had a significant impact on a PE:PC ratio and membrane stability. On the other hand, a decrease of some PC lipid species during re-hydration in both genotypes was probably associated with their metabolic functions, as they could apply to the biosynthesis of other type of lipids (Fan et al., 2013).

The functions of PS in plant cells are not well-recognized and these compounds are present there in only minor amounts, and probably their location is restricted to an inner surface of plasma membrane (Vance and Steenbergen, 2005). It is worth mentioning that the PS species are also associated with biosynthesis pathways of PE and PC (Larsson et al., 2006). Herein, mostly the PS species with very long FA chains were observed. It has been suggested that those lipids could be associated with a vesicular transport and a regulation of bilayer membrane curvature (Vincent et al., 2001). The results of PS accumulation levels in our introgression forms, presented herein, revealed that the most significant differences were observed on the third day of drought with a higher accumulation of particular PS species in the genotype 4/10. This may be associated again with a faster response of this genotype to drought conditions. However, no important differences between the genotypes after 11 days of drought, and after re-hydration were observed, indicating rather no significant impact of PS accumulation on the response to drought and re-hydration in the analyzed genotypes.

Phosphatidylinositol is an important membrane component associated with a signaling pathway and a regulation of cell metabolism in response to environmental stress factors, initiating a cascade of signals to activate the genes responsible for a plant's adaptation to adverse conditions, including drought (Xue et al., 2009). It is also a precursor in biosynthesis pathways of other phospholipids (Löfke et al., 2008). The overexpression of PI synthase gene in maize and *Nicotiana tabacum* (tobacco) increased the accumulation levels of phospholipids from PI, PC and PE classes, as well as galactolipids from the MGDG and DGDG classes. This was positively correlated with a higher level of membrane regeneration during recovery after stress cessation in the transgenic plants, compared to the wild forms (Zhai et al., 2012; Liu et al., 2013). The elevated accumulation level of PI lipid species in the genotype 4/10 observed herein could also be associated with a more efficient stress signaling pathway, and supports the other results obtained herein for the MGDG, PG, and PE pools indicating earlier and stronger response of 4/10 genotype to the stress, compared to the genotype 7/6, which showed a more gradual response. Moreover, this also supports the results regarding a faster return of the most lipid species accumulation levels to the control values in the 4/10 genotype. Lyso-phospholipids were shown to be the other compounds involved in stress signaling and stress signal transduction pathways (Okazaki and Saito, 2014). Herein, for example, higher accumulation levels of Lyso-DGDG 18:3 and Lyso-MGDG 18:3 species were observed on the first days of drought treatment in the genotype 4/10, and their abundance was shown to be significantly higher, compared to the control conditions for both analyzed genotypes (Figure 4).

### A metabolic lipid composition

Diacylglycerols are transient lipids playing a main role in the synthesis of other lipids (Cagliari et al., 2011). These lipids can be used directly for *de-novo* synthesis of TAG lipids, phospho- and galactolipids or can be converted to a phosphatidic acid, which is subsequently inserted into a phospholipid biosynthesis pathway in endoplasmic reticulum, and galactolipid biosynthesis in a chloroplast envelope (Larsson et al., 2006; Fan et al., 2013). Depletion of DAG during a drought period in both introgression forms, and a subsequent increase of their abundance after re-watering, are consistent with previously reported data (Gasulla et al., 2013).

The FAs are accumulated in plants in the oil bodies in the form of triacylglycerol lipids in storage organs, such seeds or fruits. It was reported, however, that some plant species could store the TAG lipids in other types of organs such as stems, roots or leaves, although the TAG accumulation in leaf cells is not a common process (Lin and Oliver, 2008). It was proposed that the TAG accumulated in vegetative tissues could have two primary functions. First, they can serve as a form of energy reservoir and second, as a protection against a toxicity of free FAs (Fan et al., 2013). The results obtained herein show that leaves of forage grasses can accumulate the carbon also in a form of TAG species. This result may, to some extent, explain a very low starch content in leaves of forage grasses (Cairns, 2002 and also our unpublished data). The increased accumulation level of TAG was observed earlier e.g. in *Gossypium hirsutum* L. (cotton) leaves exposed to dehydration conditions (Pham Thi et al., 1989), and during desiccation in *C. plantagineum* (Gasulla et al., 2013). The research on *Malus* (crabapple) hybrid plant leaves revealed that the TAG accumulation levels increased during the day and decreased at night (Lin and Oliver, 2008). Thus, it was suggested that TAG accumulation was associated with a photosynthesis capacity of analyzed plant species. Interestingly, the accumulation of higher amount of TAG pool during drought in the current paper was in fact observed in the genotype 7/6, which had revealed a higher photosynthetic capacity on the 11th day of drought, compared to the genotype 4/10 (Perlikowski et al., 2014). However, the precise functions of TAG during abiotic stresses, including drought conditions have yet to be recognized in plants. It cannot be excluded that the accumulation of TAG could be, at least partially, a result of nitrogen deprivation (e.g., Levitan et al., 2015) during drought conditions. A lower level of water uptake during the first days of water deficit was observed in the genotype 4/10, and this could, in fact, influence nutrient uptake (Perlikowski et al., 2014). That genotype simultaneously revealed a lower TAG abundance, compared to the 7/6 genotype, but accumulated a 2-fold higher amount of TAG during drought and re-hydration, compared to the control (Supplementary Table S4).

## Conclusions

The results presented here clearly show that water deficit has a significant impact on lipid metabolism in forage grasses. Both structural and metabolic lipid species changed their abundance during drought and subsequent re-watering periods. Some crucial genotype-dependent relations were also revealed. Moreover, several changes in the analyzed lipid profiles observed during the experiment could be associated with the physiological parameters described in detail for the analyzed genotypes. For example, most of the chloroplast membrane lipids and many extra-chloroplast lipid species, as well as DBI for most lipid classes, revealed a faster response to drought conditions in the genotype 4/10, compared the genotype 7/6. These lipids started to increase their abundance on the third day of stress treatment in the 4/10 and this could be associated with more efficient stress signaling pathways in that genotype. Furthermore, after re-hydration, the genotype 4/10 showed a significant reduction of most chloroplast lipids accumulation levels, compared to the 11th day of drought. This could be associated with a greater capacity of that genotype to regenerate after re-watering, and to repair damaged cellular membranes. The greater abundance of TAG lipids during drought conditions in the genotype 7/6 was likely associated with its greater photosynthetic capacity in drought conditions, compared to the genotype 4/10. Interestingly, the revealed lipid accumulation profiles for both genotypes showed that even closely related *Lolium-Festuca* introgression forms demonstrated significant differences in a lipid metabolism during drought conditions.

## Author contributions

DP, AK designed the experiments. DP, SK, and ÄE conducted the experimental work. DP, AK, and MR drew main conclusions. AS, PK, and DP carried out the statistical analysis. DP, AK prepared the first version of the manuscript, but all the authors contributed in further writing, and finally read and approved the manuscript.

### Conflict of interest statement

The authors declare that the research was conducted in the absence of any commercial or financial relationships that could be construed as a potential conflict of interest.

## Supplementary material

Supplementary Figure S1 **The accumulation levels of analyzed lipid species distributed among nine main classes at five time-points of experiment: before drought, after 3, 6, and 11 days of drought (DR), and 10 days of re-hydration (RH) in the 4/10 and 7/6 genotypes**. The bars represent a mean value (over replications) for Log_2_ transformed sum of all the lipid species mass spectra peak intensities within a particular lipid class. Error bars represent standard errors of the means. The letters indicate groups of means that do not differ significantly at a significance level of 0.05 (Fisher's LSD-test).

Supplementary Table S1 **The normalized mass spectral intensity means from four biological replicates and physical and chemical annotations for the analyzed lipid species and lipid classes**.

Supplementary Table S2 **ANOVA results for the analyzed lipid species and lipid classes**. Calculations were performed for five time-points of experiment: before drought, after 3, 6, and 11 days of drought, and after 10 days of re-hydration in the 4/10 and 7/6 genotypes, based on [Log_2_] transformed normalized mass spectra peak intensities. Lipids denoted as in Supplementary Table S1.

Supplementary Table S3 **Loadings of all the components calculated during PCA for the analyzed lipid classes, lipid species within the class, and (EL) as variables**. Calculations were performed for five time-points of experiment: before drought, after 3, 6 and 11 days of drought, and after 10 days of re-hydration in the 4/10 and 7/6 genotypes, based on normalized [Log_2_] transformed mass spectral peak intensities. Lipids denoted as in Supplementary Table S1.

Supplementary Table S4 **The relative percentage differences in accumulation levels of the analyzed lipid species and classes**. Calculations were performed for four time-points of experiment: after 3, 6, and 11 days of drought (DR), and after 10 days of re-hydration (RH) in the 4/10 and 7/6 genotypes, according to the control values, based on normalized mean mass spectral peak intensities. Lipids denoted as in Supplementary Table S1.

## Abbreviations

DAG: diacylglycerol
DBI: Double Bond Index
DGDG: digalactosyldiacyloglycerol
EL: electrolyte leakage
FA: fatty acid
MGDG: monogalactosyldiacyloglycerol
PC: phosphatidyl choline
PCA: principal component analysis
PC1: first principal component
PC2: second principal component
PE: phosphatidylethanolamine
PG: phosphatidylglycerol
PI: phosphat idylinositol
PS: phosphatidylserine
SQDG: sulfoquinovosyldiacylglycerol
TAG: triacylg lycerol.
