# Supplementary figures and images for: Remodeling of Leaf Cellular Glycerolipid Composition under Drought and Re-hydration Conditions in Grasses from the Lolium-Festuca Complex
Source: Front Plant Sci. 2016 Jul 19;7:1027. doi: 10.3389/fpls.2016.01027 (PMC4950141; doi:10.3389/fpls.2016.01027)

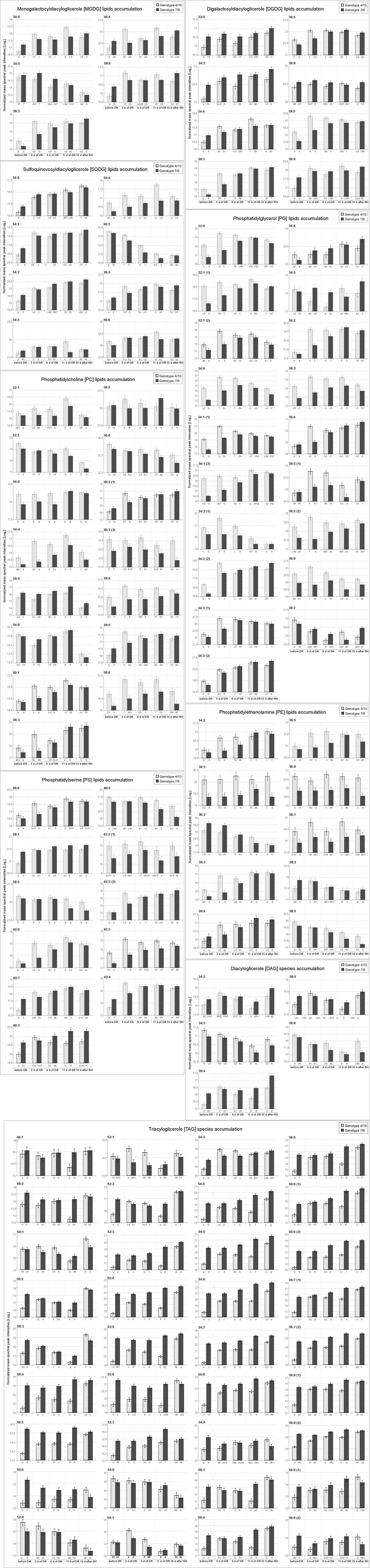

Supplement: Supplementary Figure S1 — The accumulation levels of analyzed lipid species distributed among nine main classes at five time-points of experiment: before drought, after 3, 6, and 11 days of drought (DR), and 10 days of re-hydration (RH) in the 4/10 and 7/6 genotypes. The bars represent a mean value (over replications) for Log2 transformed sum of all the lipid species mass spectra peak intensities within a particular lipid class. Error bars represent standard errors of the means. The letters indicate groups of means that do not differ significantly at a significance level of 0.05 (Fisher's LSD-test). [file Image1.jpeg]
